# Sequestration and Tissue Accumulation of Human Malaria Parasites: Can We Learn Anything from Rodent Models of Malaria?

**DOI:** 10.1371/journal.ppat.1001032

**Published:** 2010-09-30

**Authors:** Blandine Franke-Fayard, Jannik Fonager, Anneke Braks, Shahid M. Khan, Chris J. Janse

**Affiliations:** Leiden Malaria Research Group, Department of Parasitology, Center of Infectious Diseases, Leiden University Medical Center, Leiden, The Netherlands; University of California San Diego, United States of America

## Abstract

The sequestration of *Plasmodium falciparum*–infected red blood cells (irbcs) in the microvasculature of organs is associated with severe disease; correspondingly, the molecular basis of irbc adherence is an active area of study. In contrast to *P. falciparum*, much less is known about sequestration in other *Plasmodium* parasites, including those species that are used as models to study severe malaria. Here, we review the cytoadherence properties of irbcs of the rodent parasite *Plasmodium berghei* ANKA, where schizonts demonstrate a clear sequestration phenotype. Real-time in vivo imaging of transgenic *P. berghei* parasites in rodents has revealed a CD36-dependent sequestration in lungs and adipose tissue. In the absence of direct orthologs of the *P. falciparum* proteins that mediate binding to human CD36, the *P. berghei* proteins and/or mechanisms of rodent CD36 binding are as yet unknown. In addition to CD36-dependent schizont sequestration, irbcs accumulate during severe disease in different tissues, including the brain. The role of sequestration is discussed in the context of disease as are the general (dis)similarities of *P. berghei* and *P. falciparum* sequestration.

## Introduction

Erythrocytes infected with the human malaria parasite *Plasmodium falciparum* are known to cytoadhere to endothelial cells lining blood vessels, and this feature is associated with a number of features of severe malaria pathology such as cerebral malaria (CM) and pregnancy-associated malaria (PAM). This adherence of infected red blood cells (irbcs) to host tissue, also known as sequestration, occurs in small capillaries and post-capillary venules of specific organs such as the brain and lungs. Sequestration has been correlated with mechanical obstruction of blood flow in small blood vessels and vascular endothelial cell activation, which may lead to pathology [1]–[11]. As sequestration appears to be a signature of severe disease, the factors that mediate irbc adherence to endothelial cells have been the focus of numerous studies. This has resulted in the identification of parasite proteins (ligands) and host endothelium proteins (receptors, adhesins) that are directly involved in sequestration [12]–[15]. It is anticipated that increased knowledge on important features of sequestration, such as polymorphisms of receptors and ligands and their interactions, tissue distribution, affinity/avidity of binding, etc., will aid in the development of novel strategies that either reduce disease or lead to complete protection, for example through the development of vaccines or small molecule inhibitors that inhibit sequestration [8], [15]–[17].

The rodent parasite *Plasmodium berghei* is one of the most well-employed models in malaria research, and this includes analyses on the severe pathology associated with malaria infections. In particular, *P. berghei* infections can induce a number of disease states in rodents such as cerebral complications in several strains of mice [18]–[23], pregnancy-associated pathology [24]–[26], and acute lung injury [27], [28]. To what extent these different pathologies observed in laboratory animals are representative for human pathology is a matter of debate and has been discussed in a number of review papers [8], [9], [19], [21], [25], [26], [29]–[33]. Based on a number of differences in clinical features of human cerebral malaria (HCM) caused by *P. falciparum* and the cerebral pathology of *P. berghei* infections in mice, the relevance of *P. berghei* for understanding HCM has been brought into question. However, it is evident that studies on so-called experimental cerebral malaria (ECM) induced by *P. berghei* have provided insights into the critical role that a variety of host immune factors play in inducing cerebral pathology in mice. It has been argued that this knowledge may indeed be relevant for understanding, at least in part, the pathology associated with HCM, as the human condition itself is likely to represent a spectrum of pathologies. Interestingly, in contrast to the large number of studies on the role that various immune factors play in producing or mitigating *P. berghei* ECM, the role of *P. berghei* irbc sequestration in inducing these different pathologies is less well understood. In some studies it has been reported that *P. berghei* ECM is not correlated with extensive schizont accumulation in small blood vessels of the brain; cerebral complications in most ECM-susceptible mouse strains is more often associated with an accumulation of immune cells in the brain such as monocytes/macrophages, T cells, and neutrophils, and sequestration of platelets [21], [34]–[39]. In contrast, other studies have reported that *P. berghei* ECM and PAM pathology is associated with irbc accumulation in tissues such as the brain and placenta [24], [40]–[43]. In this paper, we review the available knowledge and properties of *P. berghei* irbc sequestration and show how recent advances in in vivo imaging technologies, which permit the visualization of parasite distribution and load in different organs of live mice, are being used to address issues of sequestration and disease. An understanding of *P. berghei* sequestration may help define and refine the relevance of rodent infections in understanding the different features of sequestration and pathology associated with human malaria (see Box 1 for the terminology of *P. berghei* sequestration).

**Box 1. *Plasmodium berghei* Sequestration Terminology**

**Cytoadherence of irbcs**: The specific attachment of irbcs to endothelial cells of blood capillaries and post-capillary venules, mediated by host receptor(s) and parasite ligand(s).

**Sequestration of irbcs**: An accumulation of irbcs in organs as a result of specific interactions between parasite ligands and host receptors expressed on the endothelium of blood capillaries and post-capillary venules.

**Parasite ligands**: parasite factors expressed on the surface of irbcs that mediate adherence to endothelial cells of blood capillaries and post-capillary venules.

**Host cell receptors (adhesions)**: molecules located on the surface of endothelial cells of blood capillaries and post-capillary venules that mediate sequestration of irbcs.

***P. berghei*** **CD36-mediated sequestration**: Accumulation of schizonts in organs as a result of specific interactions between the host receptor CD36 and, as yet, unknown putative parasite ligand(s).

***P. berghei*** **tissue accumulation/sequestration**: Accumulation of irbcs in organs as a result of interactions between yet unknown parasite ligands and unknown host receptors during malaria pathology (acute lung injury, ECM, and PAM).

## *P. berghei* ANKA Schizont-Infected Erythrocytes Sequester

In *P. falciparum*, the absence of mature trophozoites, schizonts, and developing gametocytes from the peripheral blood circulation of humans is clear evidence for the sequestration of these stages (Table 1). For various reasons, the detection of *P. berghei* sequestration in mice by analyzing peripheral blood or tissue histology is more complicated. Infections in mice with *P. berghei* usually result in asynchronous parasite development, which in the circulation of the host manifests itself as the simultaneous presence of different parasite life cycle stages. Characterizing parasites in peripheral blood is additionally confounded as several stages are difficult to distinguish from each other, for example young gametocytes and asexual trophozoites [44], [45]. The asynchronous course of infection in combination with the *P. berghei* schizont stage of development being relatively short (i.e., only the last 4 hours of the 22-hour erythrocytic cycle) may also hinder the detection of schizonts in excised organ tissue by histology. *P. berghei* has a strong preference for young red blood cells, reticulocytes, and these become heavily invaded both early in an infection (when the parasite tends only to infect reticulocytes) and also late in an infection (when in response to malaria anemia the host produces more reticulocytes). This often results in the presence of multiply infected reticulocytes containing up to six to eight ring forms of the parasites [46], and these cells can easily be confused with schizonts, as the irbc now also has multiple nuclei. Moreover, in *P. berghei* infections with higher parasite loads (parasitemias greater than 5%), an increased number of schizonts are found in the blood circulation. It is unknown whether the presence of circulating schizonts at high parasite densities is due to the inability of schizonts to adhere to host endothelium or is caused by factors that are related to the high parasite loads. The particular characteristics of a *P. berghei* infection, and the fact that cerebral complications are not associated with extensive schizont accumulation in the brain, has led to the common misconception that cytoadherence of *P. berghei* schizonts to host microvasculature is not as pronounced as it is in *P. falciparum*.

**Table 1:** Sequestration Properties of Blood Stages of *P. falciparum* in Humans and *P. berghei* ANKA in Rodents.

	*P. berghei* ANKA	*P. falciparum*
	**Sequestration of blood stages**	
**Stage of sequestration**	Schizonts (from the onset of nuclear division).(Maturing trophozoites do NOT sequester and all gametocyte stages appear to remain in circulation [24], [44], [47])	Maturing trophozoites, schizonts, and immature gametocytes.
**Host cell receptors for adherence**	CD36.Identified by in vivo analysis of sequestration in CD36^−/−^ mice using in vivo imaging [35].	Multiple receptors such as CD36, ICAM1, PCAM-1/CD31, CR1, CSA.Identified using in vitro binding assays [5], [8], [13].
**Parasite ligands**	Parasite ligands have not been identified. *P. berghei* does not express proteins with homology to PfEMP1 but express proteins belonging to other multi-gene families that are exported to the red blood cell [66].	PfEMP1.Identified using in vitro binding assays [63]–[65].
**Sites of sequestration**	Abundant CD36-mediated schizont sequestration in lungs and adipose tissue.Accumulation of schizonts in the spleen [35], [43].	Many different tissues, including the brain, lungs, spleen intestine, skin, fat tissue, bone marrow, and skeletal and cardiac muscles [53], [105].Organ sequestration has been assessed by examining post-mortem tissue obtained from individuals that died from severe malaria.
	**Sequestration of blood stages associated with severe disease**	
**Stage of sequestration**	Schizonts.In addition, sequestration/accumulation of other asexual blood stages [24], [41]–[43].	See above.In addition, evidence has been presented for sequestration of young trophozoites during severe disease [105], [106].
**Host cell receptors**	CD36 for schizonts (see above). Receptors for other blood stages have not been identified.	See above.During severe disease the distribution of schizont sequestration may change as a result of changes in expression of host cell receptors [107]–[110].
**Parasite ligands**	See above.No putative ligand identified for CD36-mediated sequestration nor any other ligand that may be involved in sequestration/accumulation of other blood stages during severe disease.	See above.During severe disease and pregnancy associated malaria selection favors parasites expressing specific variants of PfEMP1 [108], [111]–[113].
**Sites of sequestration**	CD36-mediated schizont sequestration in lungs and adipose tissue [35].Accumulation of schizonts in the spleen [35].Accumulation of irbcs in different tissues including the brain and placenta of pregnant mice [24], [26], [41]–[43].	See above.Abundant sequestration in brain tissue in many patients that died from cerebral complications and in the placenta of pregnant women [15], [114], [115].

**Figure 1:**
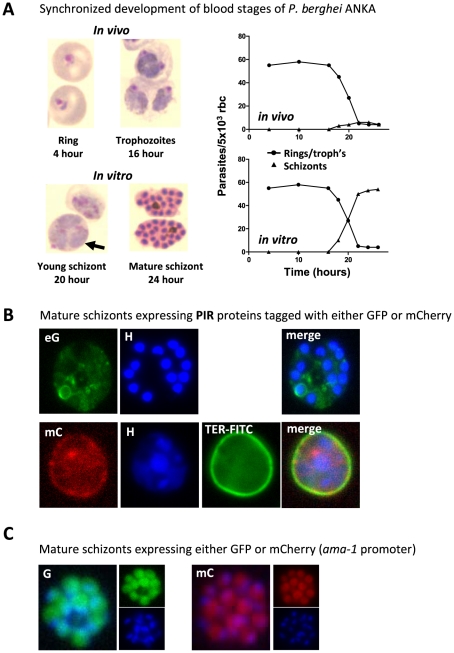
*P. berghei* ANKA asexual blood stage development and expression of proteins in mature schizonts. (A) In vivo and in vitro development of rings, trophozoites, and schizonts during one cycle of synchronized development. In mice, rings and trophozoites do not sequester but schizonts disappear from the peripheral circulation (upper graph). In vitro schizogony takes place between 18 and 24 hours after invasion of the red blood cell (lower graph). The arrow indicates a multiply infected red blood cell containing three trophozoite-stage parasites; above this cell is a 20-hour schizont (graphs adapted from [44] and [35]. (B) Live mature schizonts of two transgenic lines expressing two different fluorescently tagged PIR proteins either tagged with GFP (eG; PB200064.00.0) or mCherry (mC; PB200026.00.0). These proteins are exported into the cytoplasm of the erythrocyte nucleus stained with Hoechst (H; blue), red blood cell membrane surface protein stained in mC parasites (TER-FITC; green) (J. Braks and B. Franke-Fayard, unpublished data). (C) Live mature schizonts that express GFP and mCherry in the cytoplasm of the merozoites (J. Braks and B. Franke-Fayard, unpublished data).

Analysis of peripheral blood of experimentally synchronized infections (see Box 2) has, however, clearly demonstrated that schizonts of the *P. berghei* ANKA strain have a distinct sequestration phenotype [35], [44], [47], resulting in the disappearance of all schizogonic stages from the peripheral blood circulation. Synchronized *P. berghei* infections in mice can be established by intravenous injection of purified, fully mature schizonts [44], [47]. In contrast to most other *Plasmodium* species, viable and fully mature *P. berghei* ANKA schizonts can easily be collected and purified from in vitro cultures ([48]; Figure 1A). Injection of these schizonts into naïve mice results in a rapid release of merozoites and an almost simultaneous re-invasion of erythrocytes. In this way, synchronized infections can be established with a stable parasitemia of 0.5%–3% at 4 hours after schizont injection with the newly invaded ring forms developing into mature trophozoites within 16–18 hours. These mature trophozoites then rapidly and reproducibly disappear from the blood circulation where >90% of the parasites that demonstrate nuclear division are absent from the peripheral blood, resulting in a clear drop in parasitemia between 18 and 22 hours after schizont injection (Figure 1A). At approximately 22 hours after inoculation, the first newly invaded merozoites re-appear in the blood circulation, and subsequent invasion of merozoites gives rise to a second cycle of synchronized development [35], [44]. Quantitative analysis of the different blood stages in synchronized infections has shown that ring stages and trophozoites (up to 16–18 hours old), as well as immature and mature gametocytes, all remain in circulation [44]. These observations conclusively demonstrate that *P. berghei* ANKA schizonts are being retained in deep tissue and, moreover, that this process is very tightly regulated, as sequestration of all parasites starts with the onset of nuclear division. This absence of schizonts in peripheral blood is not only observed in experimentally induced, synchronized *P. berghei* ANKA infections but also in asynchronous infections in mice, rats, and in the natural host *Grammomys surdaster* [35]. Thus the sequestration of *P. berghei* schizonts (i.e., their absence of the peripheral circulation) is comparable to *P. falciparum* schizont sequestration. However, in *P. falciparum*, in addition to schizonts, maturing trophozoites and immature gametocytes are absent from peripheral blood, whereas there is no evidence that these stages sequester in *P. berghei* infections (Table 1). Further, in *P. berghei* infections, schizonts are more often detected in peripheral blood at higher densities, whereas in *P. falciparum* infections in humans this is rarely observed.

**Box 2. *P. berghei* ANKA Infections in Rodents**

**Synchronized infections**: Experimentally induced infections by intravenous injection of mature schizonts resulting in the synchronized development of asexual blood stage parasites for up to two developmental cycles.

**Asynchronous infections**: Infections, usually established by an intra-peritoneal injection of 10^4^–10^5^ irbcs that exhibit all parasite developmental stages in the blood simultaneously. These infections result in ECM pathology in ECM-susceptible mice, usually on day 6–8 after infection.

## *P. berghei* ANKA Schizonts Accumulate in Lungs, Adipose Tissue, and Spleen

**Figure 2:**
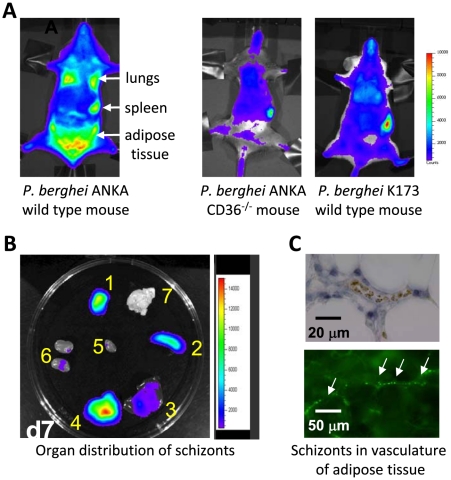
Imaging of transgenic *P. berghei* ANKA parasites in vivo and ex vivo. CD36-mediated sequestration of schizonts in adipose tissue and lungs (adapted from PNAS, 2005 [35]). (A, B) Distribution of transgenic *P. berghei* ANKA parasites, expressing GFP::luciferase fusion protein (*ama-1* promoter, see Box 3). Parasites are visible in lungs, spleen, and adipose tissue in wild-type mice, and principally in the blood circulation and accumulated in the spleen in CD36 knock-out mice. In wild-type mice infected with a non-sequestering K173 line, schizonts are also mainly found in the peripheral blood circulation and accumulated in the spleen (1: adipose tissue; 2: spleen; 3 liver; 4: lungs; 5: heart; 6: kidney; 7: brain). (C) Sequestration of transgenic *P. berghei* ANKA parasites in microvasculature of adipose tissue (upper panel with under phase contrast and lower panel with GFP-positive schizonts indicated by arrows).

A number of histological studies of animals infected with *P. berghei* have failed to detect clear sequestration of irbcs, specifically schizonts, in the brain microvasculature during ECM, raising the question, if schizonts do not sequester in the brain, where are they retained? In more recent studies in which schizonts were visualized by real time imaging in live mice, it was revealed that the lungs, adipose tissue, and the spleen are the major organs in which schizonts specifically accumulate [35], [43], [49]. Infections with transgenic *P. berghei* ANKA parasites, expressing the bioluminescent reporter-protein luciferase in conjunction with real time imaging, have shown that schizonts can be clearly discerned in these organs (see Box 3 for different transgenic parasite lines used for in vivo imaging). By introducing the luciferase gene into the genome under the control of a schizont-specific promoter (i.e., the *ama-1* promoter), only the schizont stage is made visible when detecting bioluminescence signals, and this stage of the parasite is specifically localized in lungs, adipose tissue, and the spleen (Figure 2). No significant level of schizont accumulation could be detected in other organs such as the brain, liver, and kidneys. This pattern of schizont accumulation is not restricted to experimentally induced, short-term synchronous infections; highly similar patterns of schizont accumulation have been found during asynchronous infections in both laboratory rodents and in *G. surdaster* [35]. In *P. falciparum*, sequestration in organs has mainly been assessed by examining post-mortem tissue obtained from individuals that died from malaria. These studies have revealed that *P. falciparum* schizonts sequester in differing amounts in tissues of a variety of organs (Table 1). The lung and the spleen are recognized sites for accumulation of *P. falciparum* schizonts, but adipose tissue sequestration is less reported on. However, in several studies this tissue has been identified as a site of *P. falciparum* schizont sequestration [50]–[52], [53]. What is less clear is whether the spleen in *P. berghei*–infected mice is a site of sequestration or if schizonts are trapped in the spleen as a result of selective clearance of irbcs from the blood [54]. For *Plasmodium vivax* it has been proposed that schizonts specifically adhere to barrier cells in the human spleen allowing the parasite to escape spleen-clearance while simultaneously facilitating the rapid invasion of reticulocytes [55], [56].

**Box 3. Different Luciferase-Expressing *P. berghei* ANKA Parasites That Are Used for Real Time Imaging of Parasite Distribution in Live Mice**

- ***P. berghei*** **ANKA: Luciferase expression controlled by the schizont-specific** ***ama1*** **-promoter,** **RMgm-30** and 32^*^
  Analysis of sequestration of schizonts in synchronized infections. Bioluminescence of sequestered schizonts (also newly invaded ring forms show luminescence resulting from carry over of luciferase from the mature schizont stage).
- ***P. berghei*** **ANKA: Luciferase expression controlled by the constitutive “all stages”** ***eef1a-*** **promoter,** **RMgm-28** and 29^*^
  Analysis of tissue distribution of irbcs. All stages are bioluminescent. These lines produce gametocytes that can complicate tissue distribution analyses as a result of high luminescence signals derived from circulating female gametocytes.
- ***P. berghei*** **ANKA: Luciferase expression controlled by the constitutive “all stages”** ***eef1a-*** promoter, RMgm-333^*^
  Analysis of tissue distribution of asexual blood stages. All stages are bioluminescent. This line does not produce gametocytes.
- ***P. berghei*** **K173: Luciferase expression controlled by the schizont-specific** ***ama1*** -promoter, RMgm-375^*^
  Bioluminescence of schizonts (also newly invaded ring forms show luminescence resulting from carry over of luciferase from the mature schizont stage). Schizonts of this line do not sequester and this line does not produce gametocytes.
- ***P. berghei*** **K173: Luciferase expression controlled by the schizont-specific** ***ama1*** -promoter, RMgm-380^*^
  All stages are bioluminescent. Schizonts of this line do not sequester and this line does not produce gametocytes.

*These lines have been described in the RMgm-database (http://www.pberghei.eu/) of *P. berghei* mutants.

## CD36 Is a Major Receptor for *P. berghei* ANKA Schizont Sequestration in Lungs and Adipose Tissue

Through the use of in vitro binding assays, a number of host molecules that are expressed on the surface of endothelial cells have been identified as playing a role in *P. falciparum* irbc adherence, such as CD36, ICAM-1, PCAM-1/CD31, CR1, and chondroitin sulphate A (CSA) (for review see [5], [8], [13]). Infected erythrocytes from different *P. falciparum* isolates have quite different binding affinities and preferences with respect to these host receptors [8], [57], [58]. Most *P. falciparum* isolates, however, demonstrate a capacity to bind to CD36 [58], and CD36 and CSA are the only two receptors that maintain a stable stationary adherence to irbcs.

The application of in vivo imaging techniques in laboratory animals that either express or lack CD36 revealed that CD36 also has a major role in *P. berghei* schizont sequestration, specifically in adipose tissue and lungs (Figure 1; [35]). In animals deficient in expressing CD36, the sequestration of schizonts in both the lungs and adipose tissue was strongly reduced. This was the first report, in any *Plasmodium* species, that analyzed irbc sequestration to host cell receptors in a living animal, and in real time through the course of an infection. In mice there is very little or no CD36 expression on endothelium of brain capillaries and post-capillary venules [59], [60], but there are high levels of CD36 expression on endothelium of lungs and adipose tissue [60], [61]. This sequestration is clearly consistent with schizont sequestration in the lungs and adipose tissue and absence of sequestration in the brain. Compared to the lungs or adipose tissue, *P. berghei* schizont accumulation in the spleen does not decrease in the absence of CD36 (Figure 1; [35]), and therefore accumulation in this organ results either from “nonspecific trapping” of schizonts or from interactions of schizonts to alternative host receptors. The role of CD36 as a major receptor for the adherence of *P. falciparum* irbcs to endothelial cells is well established and this receptor is known to be abundantly expressed on the surface of capillary endothelial cells in human adipose tissue [60], [61]. *S*urprisingly, however, no detailed studies have been conducted in human malaria on the extent and importance of adipose tissue. The observations of CD36-mediated sequestration of *P. berghei* and binding of irbcs of *Plasmodium chabaudi* to CD36 [62] suggest that binding to CD36 is an “ancient” feature of the *Plasmodium* genus. It would also indicate that rodent parasites modulate the surface of irbcs through the active export of proteins that contain as yet unidentified and potentially conserved receptor-binding domains (see below).

## Parasite Ligands Involved in CD36-Mediated Sequestration of *P. berghei* ANKA Schizonts Are Still Unknown

The variant protein PfEMP1 plays a major role in adherence of *P. falciparum* irbcs to different endothelial host receptors. This protein is encoded by a family of approximately 60 (*var*) genes within each parasite genome, and the expression of these proteins is mutually exclusive with only one PfEMP1 expressed on the surface of the irbc at any one time (for reviews see [63]). The extracellular region of this protein contains multiple adhesion domains, such as DBL domains, named for their homology to the EBL domains involved in red blood cell invasion, and one to two cysteine-rich interdomain regions (CIDRs). The binding domains for several host receptors have been mapped to the various DBL and CIDR domains with the CIDR1α domain mediating binding to CD36 [64], [65]. The *P. berghei* genome does not contain genes with homology to the *var* genes [66] and, as yet, no other *P. berghei* proteins have been identified that may bind to CD36. Hidden Markov Model (HMM) building using *P. falciparum* CIDR and DBL domains, followed by searches of all predicted *P. berghei* proteins available in PlasmoDB (release 6.3), have not provided evidence for the presence of proteins containing these domains (J. Fonager, unpublished data). Indeed, the only proteins in rodent malaria parasites that have been identified as being either expressed close to or on the irbc surface are members of the variant multigene family “*Plasmodium* interspersed repeats” (PIRs) [67]. These genes are shared between human, rodent, and monkey malaria species [68]–[71]. It is, however, questionable whether these proteins mediate adherence of schizonts to CD36. PIR proteins are also expressed in the human parasite *P. vivax* [56], [72], and although it has been suggested that sequestration of *P. vivax* irbcs might occur in the spleen and in other organs such as the lung during severe disease [55], [73], [74], there is no evidence for adherence of the schizonts stage to CD36 since in most infections schizonts can be found in the peripheral circulation. In addition, it is also not clear yet whether the putative extracellular domains of these proteins are indeed exposed on the outer erythrocyte membrane [56]. Analysis of the localization of two GFP- and mCherry-tagged *P. berghei* members of this family (PB200064.00.0; PB200026.00.0) showed that these proteins are exported into the host erythrocyte, but we have been unable to demonstrate an exposed surface location of these proteins ([75]; B.F. and J. Fonager, unpublished data; Figure 1B). Various other multigene families exist in *P. berghei* that have features of proteins exported into the host erythrocyte and which may modify the surface membrane of the irbc. For example, *P. berghei* orthologs to genes encoding proteins of the PYST-A and PYST-B gene families contain either predicted signal peptides only (PYST-A) or predicted signal peptide and the *Plasmodium* export (PEXEL) motif (PYST-B) [69], [76] that target proteins out of the parasite into the red blood cell, and we have indeed found that PYST-A proteins are exported into the cytoplasm of the erythrocyte (J. Braks, unpublished data). It is, however, possible that CD36-mediated schizont sequestration in *P. berghei* does not depend on the incorporation of parasite molecules onto the irbc surface but is the result of changes in the red blood cell membrane itself. For instance, the intracellular parasite may cause a disruption in the asymmetric distribution of molecules in the irbc membrane bilayer, for example, phosphatidylserine (PS). This molecule, PS, is localized on the inner leaflet of the lipid bilayer and can become “flipped” on to the outer surface of the erythrocyte under some physiological conditions. It is known that PS is able to interact directly with CD36 [77]–[79], and evidence has been found that adherence of *P. falciparum* irbcs to CD36 is in part mediated by surface-exposed PS on irbcs. Similarly, it has been proposed that *P. falciparum* is able to modify the red blood cell protein band 3, and such modifications permit irbc adherence to CD36 [13]; the parasite proteins responsible for this alteration of band 3 remain to be characterized. Clearly, further research is required to unravel the mechanisms and proteins involved that mediate CD36-sequestration of *P. berghei* schizonts. Leaving aside issues relating to cerebral complications, a greater understanding of the cellular and tissue distribution of CD36 in the host as well as the mechanisms by which this receptor is recognized by *P. berghei* parasites is likely to shed light on what are likely to be very similar processes in CD36-mediated sequestration of P. *falciparum* irbcs.

## Is CD36-Mediated Sequestration of Schizonts Associated with Cerebral Complications in Mice?

Several studies report evidence indicating that CD36-mediated sequestration of *P. berghei* is not directly associated with cerebral pathology. Firstly, infections in CD36-deficient mice exhibit no sequestration in lungs and adipose tissue but still develop ECM [27], [35]. In addition, a *P. berghei* ANKA mutant, generated by a single gene deletion, is found not to induce cerebral complications but shows a completely normal distribution of CD36-mediated schizont sequestration [43]. Lastly, ECM-susceptible mice infected with a laboratory line of the K173 strain of *P. berghei* parasites that lack schizont sequestration do develop ECM (Figure 2; [80], [81]). The absence of schizont sequestration can most likely be explained by the laboratory history of this line; it has been kept for more than 20 years in mice by mechanical blood passage and has completely lost gametocyte production and schizont sequestration, and its chromosomes are reduced in size as a result of the loss of subtelomeric genes and repeat elements ([82], [83]; J. Fonager, unpublished data). This line has frequently been used to study *P. berghei* ECM [80], [81], supporting the other published observations that schizont sequestration is not a prerequisite for *P. berghei* ECM. Interestingly, parasites of another laboratory line of the K173 strain have been shown not to induce cerebral complications [84], [85]. The sequestration pattern of the schizonts of this line is unknown, but these observations show that the capacity to induce ECM is not a stable feature of *P. berghei* strains. This has also been shown by analyzing clones of the ANKA strain that showed differences in their abilities to induce ECM [23].

The significance of studies examining *P. berghei* ECM for understanding human pathology has been brought into question, mainly because a number of differences exist in cerebral pathology between mice and humans, and also because of the observation that there appears to be a close association between the level of sequestration in the brain and HCM [33]. Therefore, the lack of an association between CD36-mediated schizonts sequestration and cerebral complications in *P. berghei*–infected mice appears to challenge the relevance of *P. berghei* as a model of HCM [33]. However, the contribution of *P. falciparum* CD36-mediated irbc adherence to human pathology is also not resolved. For example, in human infections, variation in irbc binding to CD36 has been correlated with either no effect, an increase, or a decrease in disease severity [86]–[91]. As expression of CD36 on endothelium in the brain is virtually absent, direct adherence of irbcs to endothelial CD36 is unlikely to account for significant cerebral sequestration. On the other hand, CD36 is highly expressed on monocytes, macrophages, and platelets, and it has been proposed that irbc sequestration in the brain may be mediated via irbc attachment to CD36 of sequestered platelets that act as a bridge between endothelial cells and irbcs [92], [93]. Severity of disease has also been attributed to platelet-mediated clumping of *P. falciparum* irbcs [90]. It has been argued that CD36 may also have a beneficial role in that the innate immune response may be modulated by irbc binding to CD36 on macrophages, resulting in non-opsonic phagocytosis [59], [94], [95]. A beneficial effect of CD36 expression on macrophages and dendritic cells resulting in reduced virulence has also been observed in *P. berghei* infections [96]. Moreover, adherence to CD36 expressed in microvascular endothelium of the skin and adipose tissue may reduce pathology, as it directs irbcs away from more vital and potentially life threatening sites such as the cerebral microvasculature. Although a clear association between sequestered irbcs and HCM exists in *P. falciparum*, additional investigations are required to unravel the role that CD36-dependent cytoadherence has for either pathogenesis or protection from disease in *P. falciparum* infections.

## Evidence for CD36-Independent Sequestration of *P. berghei* ANKA

**Figure 3:**
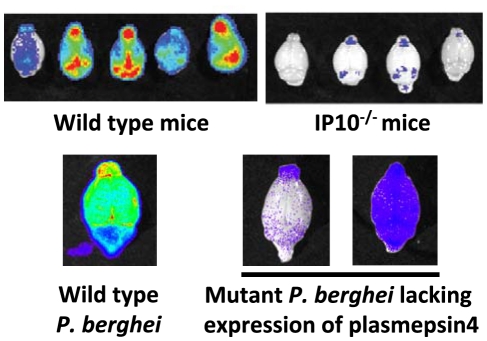
Imaging of transgenic *P. berghei* ANKA parasites in brains of mice ex vivo. Matched sets of experiments with *P. berghei* ANKA infections in ECM-sensitive mice (i.e., wild-type mice) or knock-out mice (i.e., IP10^−/−^). Knock-out mice do not develop cerebral pathology and this corresponds to a strong reduction in irbc accumulation as compared to infections in wild-type mice (adapted from [41]). Similar examples of a lack of irbc accumulation can be observed in the brains of mice treated with antibodies against host molecules (e.g., anti-LTβ mAB and anti-CD25 mAB; see [42], [104]). Parasites express GFP::luciferase fusion protein under the control of the eef1a promoter, see Box 3). Also, mice infected with a *P. berghei* ANKA mutant that has had the gene encoding plasmepsin 4 removed do not develop cerebral complications, and again there is a strong reduction of irbc accumulation in the brain of these infected animals (adapted from [43]).

The distribution of *P. berghei* schizonts in the organs in mice has principally been analyzed by the imaging of schizonts in short-term synchronous infections in living animals. Such patterns are not likely to identify alterations in sequestration due to changes in expression of other putative endothelial receptors, something that is likely to occur after the initial stage of an infection. For example, it has been shown that inflammatory markers and adhesins such as ICAM-1 become up-regulated on endothelium of the brain and lung microvasculature during *P. berghei* ECM [27], [97], [98]. When the distribution of *P. berghei* schizonts was imaged in live mice during prolonged infections, no major changes in the organ distribution of schizont sequestration were observed [35]. This would suggest that CD36 remains the major binding receptor for schizonts and that there is no obvious switch in schizont adherence phenotype during an infection. Several recent studies have shown, however, that severe disease complications such as ECM and PAM are associated with a distinct increase of irbcs accumulating in different tissues, including the brain and placenta [24], [41]–[43], indicating that additional factors play a role in irbc sequestration after the initial phase of a *P. berghei* infection. It has been shown by in vivo imaging that the timing of this irbc accumulation in the brain coincides with the development of cerebral complications, and mice protected from cerebral complications do not show a similar increase of irbc sequestration in the brain (Figure 3). In these studies the parasites used for in vivo imaging of sequestered irbcs expressed luciferase under the control of the constitutive *P. berghei* eef1a promoter (Box 3), and therefore it is not possible to discriminate between sequestered schizonts and other blood stage parasites such as rings and trophozoites. Whether this tissue accumulation of irbcs during severe disease is mediated by specific interactions between parasite ligands and endothelial receptors brain capillaries and post-capillary venules or is the result of other mechanisms (see above) of irbc trapping in small blood capillaries is unknown. For example, *P. berghei* irbcs have been observed to attach to the surface of endothelium-adherent monocytes/macrophages [21]. Evidence has also been found for irbc accumulation in capillaries as a result of adherence to sequestered platelets [37]. As increased mononuclear cell [34] and platelet sequestration [37], [39] is observed in the brain vessels of mice during ECM, monocyte/platelet-trapped irbcs may account, at least in part, for parasites present in the brain vasculature. On the other hand, *P. berghei* irbcs have been observed in close contact with the microvascular endothelium [40], indicating that irbcs may directly adhere to endothelial cells. Further analysis is required to provide an insight into both the amounts of parasites (i.e., load) that accumulate in the brain and the stage of the parasite that is found in tissue during severe disease. The generation of transgenic *P. berghei* parasites expressing different fluorescent reporter proteins (e.g., GFP and mCherry; see Figure 1C) now offers the possibility of directly visualizing interactions between host cells and irbcs in the brain of living mice by, for example, using multiphoton microscopy to perform intravital imaging [99]–[102]. An understanding of the mechanisms of CD36-independent sequestration may help to define the contribution of other host receptors to irbc adherence and the relationship with induction of pathology.

In addition to CD36-mediated schizont sequestration and irbc sequestration during ECM, evidence has also been found for adherence of *P. berghei* irbcs to CSA present on the surface of syncytiotrophoblasts in the placenta of pregnant mice. Specifically, irbcs in pregnancy-induced recrudescent infections showed an enhancement of in vitro adherence to placenta tissue with a marked specificity for CSA [26]. As with CD36 sequestration, the *P. berghei* ligands mediating adherence to CSA are also unknown.

## Conclusion

Revealing where and when *P. berghei* sequesters in a living host, by real time imaging of transgenic parasites, has opened up exciting possibilities into research looking at sequestration and the contribution this has to different aspects of malarial disease. Schizonts of *P. berghei* sequester in the body of living animals in distinct locations, and this appears to be related to the expression of host CD36 with abundant sequestration in adipose tissue and lungs. CD36-mediated schizont sequestration is not observed in the brain, but evidence has been presented for a CD36-independent accumulation of irbcs in different tissues during severe disease, including the brain. The characterization and the genetic modification of *P. berghei* ligands involved in binding to the different host receptors might offer novel possibilities in the development of small-animal models for analysis of sequestration properties of *P. falciparum* ligands that have hitherto only been examined in vitro. This could be performed by, for example, substituting *P. berghei* ligands with *P. falciparum* PfEMP-1 proteins or domains. Using in vivo imaging in conjunction with such “falciparumized” *P. berghei* parasites in mice expressing human receptors (e.g., human ICAM-1; [103]) may create an in vivo screening system for testing inhibitors that block *P. falciparum* sequestration. Despite clear differences between the rodent model and human infections, we believe enough similarities remain that justify further studies on *P. berghei* sequestration for obtaining more insight into how the malaria parasite uses sequestration to survive inside the host, how this may provoke disease, and how interventions may work.
